# ELDER MISTREATMENT AND ANXIETY SYMPTOMS AMONG U.S. CHINESE OLDER ADULTS

**DOI:** 10.1093/geroni/igz038.1007

**Published:** 2019-11-08

**Authors:** Ying-Yu Chao, Yu-Ping Chang, XinQi Dong

**Affiliations:** 1 Rutgers School of Nursing, Newark, United States; 2 School of Nursing, The State University of New York, University at Buffalo, Buffalo, New York, United States; 3 Rutgers Institute for Health, Health Care Policy and Aging Research, New Brunswick, United States

## Abstract

Purpose: Elder mistreatment is a critical public health issue across all cultural backgrounds. However, we have limited understanding of the mental health outcomes of elder mistreatment among older Chinese Americans. This study aimed to examine the association between different types of elder mistreatment and anxiety symptoms among U.S. Chinese older adults. Methods: Data were from the Population Study of Chinese Elderly in Chicago (PINE), a community-based participatory research study of 3,157 Chinese older adults in the Greater Chicago area. Elder abuse was assessed with the overall abuse screening test and Modified Conflict Tactic Scale. Anxiety symptoms were assessed by the Hospital Anxiety and Depression Scale (HADS‐A). Multivariate logistic regression analyses were performed. Results: Participants had a mean age of 72.8 ± 8.3 years old (range 60-105). After controlling for potential covariates, participants with overall abuse (OR = 1.76, 95% CI: 1.57-1.98, p < .001), psychological abuse (OR = 1.76, 95% CI: 1.53-2.02, p < .001), physical abuse (OR = 1.56, 95% CI: 1.03-2.36, p < .05), and financial exploitation (OR = 1.24, 95% CI: 1.06 – 1.44, p < .001) were more likely to have anxiety symptoms. However, sexual abuse was not significantly associated with anxiety symptoms (p = 0.83). Conclusion & Implications: Our findings indicate that elder mistreatment may result in anxiety symptoms among Chinese older adults. Further studies can examine whether Chinese cultural traits (e.g., face, harmony) influence the relationships between elder mistreatment and anxiety symptoms.

